# Photobiomodulation prevents PTSD-like memory impairments in rats

**DOI:** 10.1038/s41380-021-01088-z

**Published:** 2021-04-15

**Authors:** Yong Li, Yan Dong, Luodan Yang, Lorelei Tucker, Xuemei Zong, Darrell Brann, Michael R. Hamblin, Almira Vazdarjanova, Quanguang Zhang

**Affiliations:** 1 Department of Neuroscience and Regenerative Medicine, Medical College of Georgia, Augusta University, Augusta, GA USA; 2 Laser Research Centre, Faculty of Health Science, University of Johannesburg, Doornfontein, South Africa; 3 Charlie Norwood VA Medical Center, Augusta, GA USA; 4 Department of Pharmacology & Toxicology, Medical College of Georgia, Augusta University, Augusta, GA USA

**Keywords:** Depression, Neuroscience

## Abstract

A precise fear memory encoding a traumatic event enables an individual to avoid danger and identify safety. An impaired fear memory (contextual amnesia), however, puts the individual at risk of developing posttraumatic stress disorder (PTSD) due to the inability to identify a safe context when encountering trauma-associated cues later in life. Although it is gaining attention that contextual amnesia is a critical etiologic factor for PTSD, there is no treatment currently available that can reverse contextual amnesia, and whether such treatment can prevent the development of PTSD is unknown. Here, we report that (I) a single dose of transcranial photobiomodulation (PBM) applied immediately after tone fear conditioning can reverse contextual amnesia. PBM treatment preserved an appropriately high level of contextual fear memory in rats revisiting the “dangerous” context, while control rats displayed memory impairment. (II) A single dose of PBM applied after memory recall can reduce contextual fear during both contextual and cued memory testing. (III) In a model of complex PTSD with repeated trauma, rats given early PBM interventions efficiently discriminated safety from danger during cued memory testing and, importantly, these rats did not develop PTSD-like symptoms and comorbidities. (IV) Finally, we report that fear extinction was facilitated when PBM was applied in the early intervention window of memory consolidation. Our results demonstrate that PBM treatment applied immediately after a traumatic event or its memory recall can protect contextual fear memory and prevent the development of PTSD-like psychopathological fear in rats.

## Introduction

Experiencing a traumatic event leads to the formation and then consolidation of several types of fear-influenced memories, including context-associated and cue-associated memories, each of which triggers the expression of fear [1, 2]. While creating and retrieving these memories is essential for avoiding danger and identifying safety [3, 4], an improper balance in the strength of these memories can lead to psychopathological fear states [1, 5]. Individuals with developing posttraumatic stress disorder (PTSD) are well-known for having an intrusive cued memory (cued hypermnesia) that paradoxically coexists with an impaired contextual memory (contextual amnesia) [5–9]. Contextual amnesia indicates that individuals developing PTSD cannot remember the dangerous context where and when the traumatic event originally occurred [1]. In contrast, cued hypermnesia indicates that individuals developing PTSD cannot correctly identify the trauma-associated cue in a safe context after memory recall [1, 10]. Despite living in a safe environment, repeated encounters with trauma-associated cues lead to cued memory retrieval outside of the dangerous context, and eventually, the cued memory becomes pathogenic [1, 11, 12]. This implies that protecting the integrity of the contextual memory encoding the dangerous context is the key to the differentiation of a safe context (referred to as context discrimination), thereby inhibiting cued-elicited fear in the safe context and facilitating the process of fear extinction, a weakened fear response following non-reinforced exposure to a conditioned stimulus [13].

The process of context discrimination depends on hippocampal input [14, 15] because the hippocampus is essential for the formation and recollection of episodic and contextual memories of past events [11, 16, 17]. The inability to appropriately perform context or cue discrimination in animals is referred to as PTSD-like memory impairments and has been manifested previously in rodent models [1, 18]. Robust evidence has confirmed that stress hormones, including norepinephrine and glucocorticoids, released shortly after experiencing a traumatic event, can facilitate memory consolidation and form long-term memories [4]. However, the high level of corticosterone in the hippocampus, originating either from external perfusion or prolonged restraint following fear conditioning training with strong foot shock, is considered an inducer of contextual amnesia [1, 18]. Although contextual amnesia is gaining attention as a critical etiologic factor for PTSD, there is no treatment currently available that can reverse contextual amnesia. Whether such treatment can prevent the development of cued hypermnesia is unknown.

Photobiomodulation (PBM) is a non-invasive physical therapy in which low energy near-infrared light is applied to a tissue of interest and has been demonstrated to produce complex treatment effects for diverse diseases, including several brain disorders [19, 20]. PBM has also been investigated as a potential treatment for psychiatric disorders, both in animal studies and human pilot trials. Using a prolonged stress model to induce a PTSD-like phenotype in rats, the application of PBM to acupoints in both legs reduced anxiety-like symptoms and increased neural activation at the anterior cingulate cortex [21]. In two clinical pilot trials, transcranial PBM reduced depressive symptoms and demonstrated safety in human patients with major depressive disorder [22, 23]. In a study examining the effects of transcranial PBM on generalized anxiety disorder, patients treated with PBM displayed significant reductions in measures of anxiety symptoms and had improvements in their quality of sleep [24]. It is thought that the underlying mechanism mediating these effects is, in part, related to the ability of PBM to promote mitochondrial function and beneficial trophic factor release by way of modulating cytochrome c oxidase activity [25].

Cytochrome c oxidase has been identified as the primary target of PBM treatment [19, 26, 27]. Photons from PBM can dissociate inhibitory nitric oxide from cytochrome c oxidase, leading to increased electron transport across the mitochondrial inner membrane, resulting in boosted adenosine triphosphate (ATP) production [26, 27]. PBM can increase ATP production in diverse tissues [28, 29]. ATP is the primary energy supply for not only basal metabolic activities but also the memory formation process in neurons [30]. It has been shown that metabolic agents that enhance ATP can improve cognition [31]. This energy-boosting effect from PBM inspired our hypothesis that PBM treatment could protect contextual memory from impairment, increase the contextual discriminability of a safe environment, and prevent the development of PTSD-like symptoms and comorbidities in rats.

## Materials and methods

### Animals

Adult (3–5 months old) Sprague–Dawley rats of both sexes were included in this study. For the experiments that include animals of both sexes, 40–60% of rats in each group were females in order to exclude the bias induced by sex differences. Animals were bred in our campus animal facility and were group-housed (2–4 rats/cage from the same litter) in a temperature- and light-controlled room (23 °C with a light/dark cycle of 6 a.m./6 p.m.). Litter-mates from the same sex were randomly assigned to different treatment groups. Typically the treatment group and its control group had 8–15 rats. All experiments were conducted in accordance with the animal use protocol that was approved by the Institutional Animal Care and Use Committee of Augusta University.

### PBM treatment and restraint

The laser setup used in this study was based on our previous work [32] with improvements. The detailed procedure for PBM application is illustrated in Supplementary Fig. S1. The wavelength of the CW laser (PSU-III-LED, model 808M100, Dragon Lasers, Jilin province, China) at 808 nm was selected because it is a near-infrared light with the features of minimal thermal effects, deep penetration, and minimal absorption through tissue media [33]. All rats were shaved at least two days before applying PBM treatment. During treatment, rats were restrained in a transparent plastic cone (DecapiCone, DCL-120, Braintree Scientific, Inc., MA). Eyes were covered with aluminum foil in order to eliminate the interference from the visible guide light (red). The laser beam spot (808 nm, invisible to both humans and rats) was positioned using the red guide light which allows it to target the scalp easily. The distance between the end of the beam fiber lens (Imeter 400 µm multi model; adjustable focus) and the rat scalp was adjusted to 35 cm to generate a 1.5 cm^2^ laser spot size on the scalp.

To measure the beam intensity that penetrated into specific brain tissues, rats were decapitated, and the brains were dissected out by breaking the basion bone, which left the top of the skull intact. According to the rat brain atlas, horizontal brain sections were prepared from ventral to dorsal until the amygdala was first observed. Then, the remaining parts of the brain were placed back under the skull. The scalp and the plastic cone were placed on top of the skull. The laser meter probe (#LP1, Sanwa, Japan; #FM33-056, Coherent Inc, USA) was placed under the brain section (Supplementary Fig. S1d). The beam intensity that penetrated to the dorsal hippocampus and cortex was measured following the same procedure as the amygdala with smaller pieces of dissected brain tissues. The reading on the laser meter probe was 25 mW/cm^2^ for the cortex, 12 mW/cm^2^ for the hippocampus, and 3 mW/cm^2^ for the amygdala.

We have tried three different time durations of PBM treatment of 1, 2, and 4 min following the repeated fear conditioning training protocol. Our data demonstrate that 2 min treatment duration can generate the optimal beneficial effects on PTSD symptoms (data not shown). Therefore, we went with 2 min treatments throughout the rest of the study. We compared local temperature on the rat scalp after PBM treatment or restraint, using an infrared thermometer (NUB8380, Nubee). At room temperature (25 °C), we observed a 1 °C temperature increase on the scalp of living rats following 2 min of 350 mW/cm^2^ PBM treatment on the scalp (31.79 ± 0.17 °C for Res-group, *n* = 11; 32.79 ± 0.13 °C for PBM-group, *n* = 10). However, when we used the same setting as described in Supplementary Fig. S1d and monitored the temperature increase on the brain tissue after PBM treatment, we could not detect temperature changes on the cortex when compared with the tissue temperature prior to PBM treatment. Therefore, PBM inevitably imparts a thermal effect on the scalp, but it is unlikely that the PBM can generate thermal effects on brain tissue.

### Behavior tests

Based on our previous protocols [32, 34, 35], behavior tests were performed between 3 p.m. and 6 p.m. (light of at 6 p.m.) and analyzed by “ANY-Maze” software (Stoelting Co., Wood Dale, IL, USA). Data collection and analysis were performed blind to the experimenter.

### Fear conditioning

The fear conditioning chamber (context-A) is equipped with steel rods on the floor for delivering electric shocks controlled by a stimulus isolation unit (SIU-102B, Warner Instruments, Hamden CT, USA). Freezing behavior (no noticeable movement for ≥1 s) was analyzed by “ANY-Maze” software. After rats were placed in the conditioning chamber for 90 s, three 30 s tones, each of which co-terminated with a 2 s foot shock, were presented every 2 min. The percentage of freezing for every 2 min conditioning session was plotted as conditioning response. Tones were 2.8 kHz sine waves (90 dB).

### Contextual memory test

Contextual fear memory was tested in context-A for 5 min without tone presentation. The percentage of freezing time (1–5 min) was plotted to evaluate contextual memory.

### Cued memory test

Cued fear memory was tested in a different chamber named context-B (for a different visual context) with sawdust bedding (for different odor). A rat was placed in context-B for 90 s and then presented with three 30 s tones every 2 min (the same as conditioning training without foot shock). The percentage of freezing before the first tone presentation was calculated as baseline freezing to the neutral context-B, and the average percentage of freezing to three tones was plotted as cued memory.

### Memory recall test

Rats were pre-exposed to context-A for 5 min at day 0, then were conditioned (three 1.5 mA foot shocks) in context-A on day 1. On day 2, a 2 min reminder session (rats were placed in the context-A chamber for 90 s, then a 30 s tone was presented) was introduced. Immediately after the reminder tone, rats were restrained either with or without PBM treatment. Rats were then returned to their home cage. Two weeks later, on day 16, contextual memory in context-A (5 min) and cued memory (2 min, accompanied with a 30 s tone at the end) in context-B were measured.

### Extinction test

Rats were conditioned (three 0.5 mA foot shocks per day) in context-A on days 1–2, followed by extinction training (cued memory test in context-B) on days 3–6. During extinction training, rats were placed in the extinction chamber (context-B) for 90 s and then presented with three 30 s tones (90 dB) every 2 min (the same tone as conditioning training without foot shock). The decrease of freezing to tone was interpreted as an extinction effect.

### Corticosterone detection

Rats were anesthetized by inhalation of isoflurane and cardiac perfusion with ice-cold PBS. The plasma and dorsal hippocampus were collected quickly and tested for corticosterone using an ELISA kit (EIACORT; Invitrogen, Carlsbad, CA, USA). Blood was collected in a 1.5 ml tube containing 25 mg EDTA and then centrifuged at 2000 rpm for 10 min. According to the manual, 0.5 ul plasma per well was assayed for ELISA. The dorsal portion of the hippocampus was homogenized in ice-cold lysis buffer (50 mM HEPES, pH 7.4, 150 mM NaCl, 12 mM beta-glycerophosphate, 1% Triton) with a protease inhibitor cocktail (Sigma-Aldrich, Allentown, PA, USA). The lysis was then sonicated for 30 s and centrifuged at 12000 rpm for 10 min at 4 °C. The protein concentration was measured using a BCA protein assay kit (Thermo Scientific; Guilford, CT, USA), and 5 µg of total protein from hippocampus lysis samples per well was aliquoted for ELISA. The final concentration of corticosterone from plasma and the hippocampus was calculated according to the standard curve.

### Norepinephrine detection

The same tissue lysate from the above preparation for corticosterone measurement was used for norepinephrine detection by the ELISA method (KA1891; Abnova Corporation, Taipei, Taiwan). Total 300 µg protein lysis and 100 µL plasma were assayed for measuring norepinephrine according to the manual.

### ATP detection

The hippocampal lysate from the above preparation for corticosterone measurement was used for ATP detection (Firefly Luciferase Bioluminescence Assay, Invitrogen; Guilford, CT, USA). A 5 µg aliquot of the total protein sample was assayed for ATP content according to the manual. ATP concentration was quantified according to a standard curve.

### Statistics analyses

All data were presented as mean±S.E.M. Statistical comparisons between different groups across multiple consecutive time points (days, minutes) were analyzed with two-way ANOVA using SigmaStat 3.5 software. After ANOVA, Student–Newman–Keuls method post hoc tests were used for pairwise comparisons between two groups. Other comparisons between two groups were analyzed via student’s *t*-test (two tailed). A level of *P* < 0.05 was considered statistically significant. Detailed methods of statistical analyses method are listed in supplementary materials as Supplementary Table S1.

## Results

### Contextual amnesia is prevented by applying PBM immediately after fear conditioning training

**Fig. 1:**
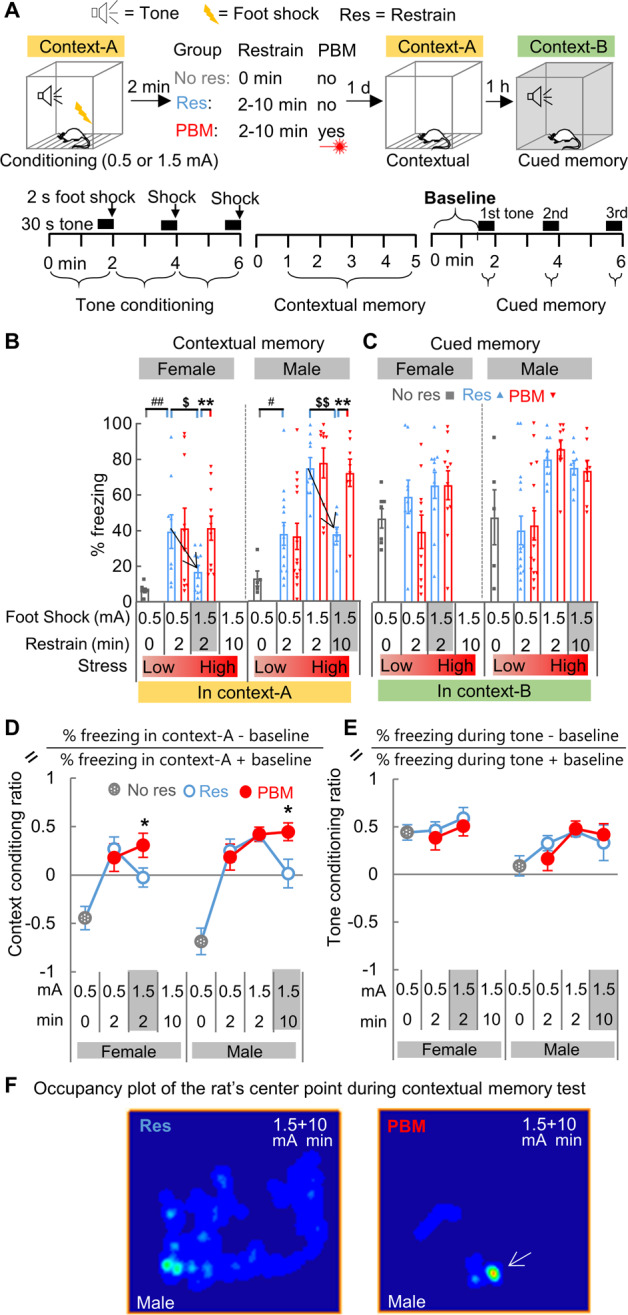
Contextual amnesia is prevented by applying PBM immediately after fear conditioning training. **A** The behavioral test paradigm for contextual amnesia. **B** As stress intensity rises, the strength of contextual memory increases. Contextual memory was impaired after a turning point, indicated by a black arrowhead, from 0.5 + 2 (abbreviation of 0.5 mA foot shock followed by 2 min restraint) (Res, *n* = 9; PBM, *n* = 10) to 1.5 + 2 (Res & PBM, *n* = 11 each) for females (^$^*P* < 0.05, *t*-test, 0.5 mA vs. 1.5 mA) and from 1.5 + 2 (Res & PBM, *n* = 9 each) to 1.5 + 10 for males (Res & PBM, *n* = 8 each) (^$$^*P* < 0.01, *t*-test, 2 min vs. 10 min). PBM treatment prevented such memory impairment in both sexes (**P < 0.01, *t*-test, PBM vs. Res). A group of rats (Females, *n* = 7; Males, *n* = 5), named as No res-group, was fear-conditioned but did not receive restraint. #*P* < 0.05, ##*P* < 0.01, *t*-test, Res vs. No res. **C** Cued memory was not impaired and unaffected by PBM. **D** The context conditioning ratio was calculated for all groups with different stress levels. **E** The tone conditioning ratio. **F** Representative occupancy plot of the animal’s center point for males of Res-group and PBM-group during contextual memory testing at stress intensity 1.5 + 10. Compared with Res-group, rats in PBM-group exhibited a much shorter exploration track and higher freezing behavior (white arrow denotes a high-occupancy location), which indicates a preserved contextual memory after PBM treatment. Data are mean ± S.E.M.

It has been established that increasing stress intensity by applying restraint immediately after fear conditioning with a strong foot shock induces contextual amnesia [1]. Here, we first attempted to create an impaired contextual memory in rats and tested whether PBM could protect it. As females have a twofold higher risk of developing PTSD than males [36], both sexes were included. Twelve groups of rats were conditioned with either three mild (0.5 mA) or strong (1.5 mA) foot shocks and accompanying tones in context-A (Fig. 1A), then were restrained for 0, 2, or 10 min immediately after conditioning. Contextual and cued memory was tested 24 h later in context-A and context-B, respectively. We established Res-group as the control group for PBM treatment because rats must be transiently restrained to administer PBM treatment. To be noted, restraint applied immediately after fear conditioning acts as a second stressor and has been reported as a critical factor that can interfere with the fear-conditioning event’s memory consolidation process [1]. Therefore, we also set up a No res-group as a reference for adaptive fear memory, and rats in this group were not restrained following fear conditioning. No res-group’s total stress intensity is abbreviated as 0.5 + 0 (0.5 mA foot shock followed by 0 min restraint). Compared with No res-group (0.5 + 0), a much higher freezing percentage was observed in Res-group (0.5 + 2) during the contextual memory test (Fig. 1B) (*t* (14) = 3.036, *P* = 0.0089 for females; *t* (17) = 2.218, *P* = 0.0405 for males), which suggests that restraint stress applied immediately after foot shock can greatly enhance contextual memory.

Interestingly, when stress intensity was increased to 1.5 + 2, the same high-stress intensity induced a contextual memory impairing effect (Fig. 1B downward arrowhead) in the females of Res-group (lower freezing at 1.5 + 2 compared with 0.5 + 2, *t* (18) = 2.411, *P* = 0.027), but a contextual memory-enhancing effect in the males of Res-group (higher freezing at 1.5 + 2 compared with 0.5 + 2, *t* (21) = 3.935, *P* = 0.0008). This result indicates that contextual fear memory in females is more prone to damage than in males. When the stress condition was further increased to 1.5 + 10, contextual memory impairment (Fig. 1B downward arrowhead) was observed in the males of Res-group (1.5 + 10 vs. 1.5 + 2, *t* (15) = 5.073, *P* = 0.0001). Strikingly, PBM prevented memory impairment in both females (*t* (20) = 3.196, *P* = 0.005, PBM vs. Res) and males (*t* (14) = 4.006, *P* = 0.0013) (Fig. 1B). Unlike contextual memory, cued memory was not impaired, and PBM had no treatment effects (Fig. 1C).

The percentage of freezing response during contextual and cued memory tests represents a summated fear response that includes not only the fear response elicited by the memory to the conditioned context and tone, but also a basal emotional fear state [37]. The basal emotional state could be monitored by recording baseline freezing (Fig. 1A lower right corner), the freezing response prior to tone presentation in a novel context. By subtracting the baseline, the conditioned fear response elicited by the conditioned tone can be used as a numerator for calculating the tone conditioning ratio [38]. In this study, the tone conditioning ratio was calculated as [% freezing during tone presentation − baseline]/[% freezing during tone presentation + baseline]. Using similar logic, the context conditioning ratio was also calculated as [% freezing during contextual memory test − baseline]/[% freezing during contextual memory test + baseline]. The context conditioning ratio reflects the context discriminability of the conditioned context-A against the novel context-B. From these new calculations, we found that PBM can prevent the dropdown of context conditioning ratio (Fig. 1D) in both sexes when stress intensity is high (*t* (20) = 2.098, *P* = 0.049, PBM vs. Res at 1.5 + 2 for females; *t* (14) = 2.468, *P* = 0.027, PBM vs. Res at 1.5 + 10 for males). The tone conditioning ratio remains stable (Fig. 1E), and PBM has no effect.

By choosing female animals, we compared the treatment efficacy of 6 h delay (6 h post-group) with immediate (PBM-group) or no treatment (Res-group) group (Supplementary Fig. S2). We found that PBM’s protective effect is compromised when PBM is postponed to 6 h outside of the consolidation window. Taken together, these results suggest that (I) applying restraint immediately after fear conditioning training with strong foot shock can disrupt the memory consolidation process and induce contextual amnesia, and (II) PBM treatment applied during restraint can preserve the consolidation process and protect contextual memory from impairment.

### PBM increases ATP in the hippocampus but has no effect on corticosterone under high-stress conditions

**Fig. 2:**
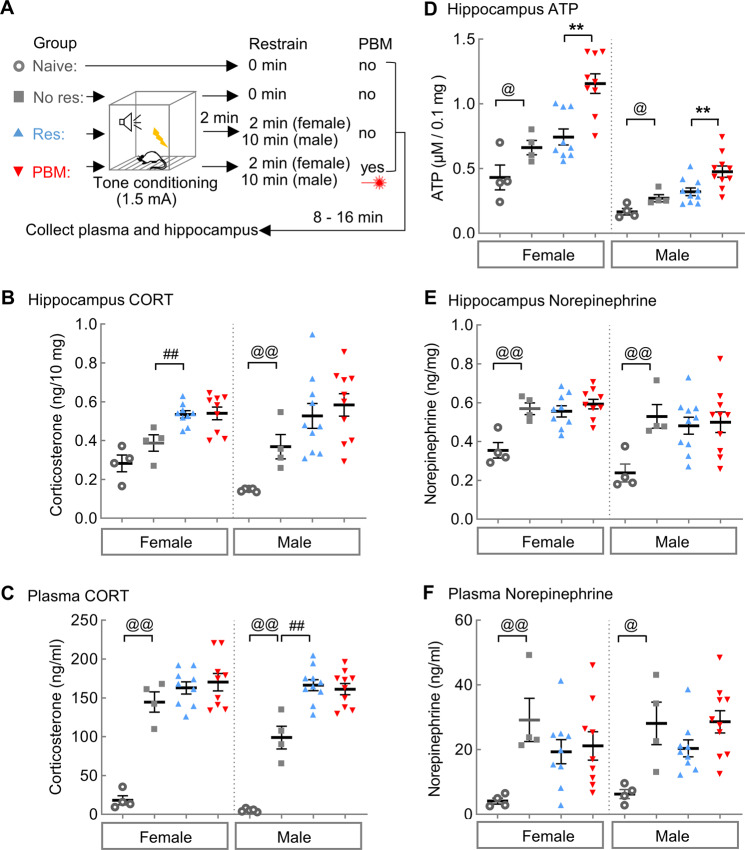
PBM increases ATP, but has no effects on the toxic corticosterone (CORT) in the hippocampus following stress which could induce contextual amnesia. **A** The stress conditions for inducing contextual amnesia are 1.5 + 2 for females (Res & PBM, *n* = 9 each) and 1.5 + 10 for males (Res & PBM, *n* = 10 each). The stress conditions are 1.5 + 0 for No res-group (females & males, *n* = 4 each) and 0 + 0 for Naïve-group (females & males, *n* = 4 each). Naïve-group rats were sacrificed at their home cage. Other rats were sacrificed at 20 min after the last foot shock. **B** Females had higher basal levels of CORT in the hippocampus than males (*t* (7) = 3.584, *P* = 0.009). Fear conditioning increased hippocampal CORT only in males (*t* (7) = 4.034, *P* = 0.005), not females (*t* (6) = 1.744, *P* = 0.132). The restraint increased CORT in the female hippocampus (*t* (11) = 3.791, *P* = 0.003), not males (*t* (12) = 1.445, *P* = 0.174). Compared with Res-group, PBM had no effect on either plasma CORT (females, *t* (16) = 0.532, *P* = 0.602; males, *t* (18) = 0.507, *P* = 0.618) or hippocampal CORT (females, *t* (16) = 0.0639, *P* = 0.95; males, *t* (18) = 0.662, *P* = 0.517). ^##^*P* < 0.01, No res vs. Res; ^@@^*P* < 0.01, Naïve vs. No res. **C** Females had higher basal level of circulating CORT in plasma than males (*t* (7) = 0.2552, *P* = 0.038). Fear conditioning greatly increased plasma CORT in both sexes (Naïve vs. No res; females, *t* (6) = 8.879, *P* < 0.001; males, *t* (7) = 7.297, *P* < 0.001). The res*t*raint further increased CORT in males (*t* (12) = 4.633, *P* < 0.001), but not females (No res vs. Res; *t* (11) = 1.254, *P* = 0.236). **D** PBM increased ATP production in the hippocampus of both females and males. **E**, **F** PBM has no effect on norepinephrine in either the hippocampus or plasma. **P < 0.01, PBM vs. Res, Student’s *t*-test. Data are mean ± S.E.M.

To test whether PBM influences the levels of corticosterone in the hippocampus, rats were conditioned with the high-stress intensity conditions, as described above, which previously induced contextual amnesia (1.5 + 2 for females; 1.5 + 10 for males). Rats were sacrificed 20 min after fear conditioning when systemic corticosterone levels reached their highest plateau (Fig. 2A). Despite a stark increase in corticosterone observed in both plasma and the hippocampus (*t* (11) = 6.406, *P* < 0.0001 for females, Res vs. Naive; *t* (13) = 4.161, *P* = 0.0011 for males) of Res-group rats compared to Naïve-group rats, PBM did not exhibit a treatment effect on the high levels corticosterone in either plasma or the hippocampus (Fig. 2B, C) (*t* (16) = 0.1352, *P* = 0.8942 for females, PBM vs. Res; *t* (18) = 0.6617, *P* = 0.5165 for males). This result suggests that PBM is not directly targeting corticosterone levels, but rather it is targeting memory interference downstream of corticosterone release.

It has been proposed that glucocorticoids endanger hippocampal neurons by impairing their energy metabolism [39]. In the minutes following a stressful event, norepinephrine promotes glucose metabolism; hours into the stress response process; however, glucocorticoids act to suppress energy metabolism [39, 40]. Interestingly, when the same tissue lysate for hippocampal corticosterone measurement was assayed for ATP concentration, an increase of ATP content was observed in PBM-group compared with Res-group (*t* (16) = 3.564, *P* = 0.0026 for females; *t* (18) = 2.974, *P* = 0.0081 for males) (Fig. 2D). The increase of ATP supply following a high-stress event in PBM-group rats implies that an improved energy supply in the hippocampus during the memory consolidation window may contribute to the contextual memory protection imparted by PBM. Using the same batch of sample aliquots from corticosterone analysis, we also measured the norepinephrine concentration. We found that PBM does not affect norepinephrine in either plasma or hippocampus (Fig. 2E and F).

### PBM reduces contextual fear following memory recall

**Fig. 3:**
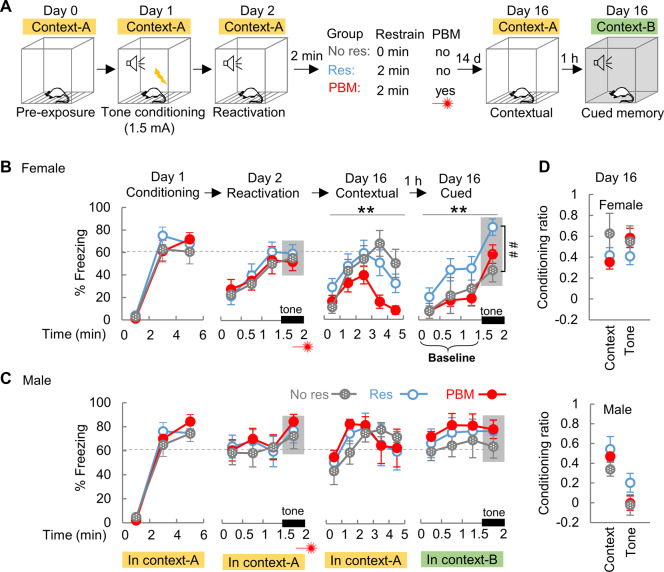
The overall contextual fear is reduced by applying PBM after memory recall. **A** The behavioral test paradigm for memory recall. **B** In female Res-group rats, a strengthened cued memory was observed at day 16 compared with No res-group (*n* = 8) (^$^*P* < 0.05, *t*-test, percentage of freezing to tone was shaded with gray as cued memory), which indicates a step towards the development of cued hypermnesia. Compared with Res-group (*n* = 10), female rats in PBM-group (*n* = 12) exhibited less freezing response during the contextual and cued memory test (**P < 0.01, two-way ANOVA with repeat measures). **C** In males (Res & PBM, *n* = 8 each), both contextual and cued memorries were intense, and PBM had no effects. The baseline is the percent of freezing before tone presentation in a novel context-B. **D** The context and tone conditioning ratio calculated at day 16 for both sexes. Data are mean ± S.E.M.

The above results demonstrate that PBM treatment applied at the consolidation window can protect contextual memory from impairment, and therefore we next investigated whether PBM has a treatment effect if PBM is applied after memory recall, another early intervention window [41, 42]. Rats were pre-exposed to context-A on day 0 (Fig. 3A), and were conditioned on day 1 (1.5 mA foot shock without subsequent restraint was chosen to generate an unimpaired contextual memory to facilitate full memory recall, according to the results in Fig. 1A). A single tone in context-A was introduced as a reminder to provoke memory recall on day 2. PBM was applied immediately after the tone reminder. Contextual and cued memory was tested on day 16 in context-A and context-B, respectively. Consistent with the sexual dimorphism observed in Fig. 1, males in Res-group exhibited a much higher freezing response to context-A than females during the 0–1.5 min time duration of the reactivation session (a shorter contextual memory test) (*t* (17) = 2.361, *P* = 0.030). During cued memory tests (Fig. 3B), female rats in Res-group exhibited higher freezing to the tone on day 16 compared with the No res-group (*t* (16) = 3.090, *P* = 0.007). This exaggerated freezing response in Res-group suggests a reconsolidation-related step towards the development of cued hypermnesia, but this phenomenon is not observed in the rats from PBM-group (*t* (18) = 1.092, *P* = 0.289, PBM vs. No res). It should be noted that rats received foot shock only on day 1, one out of four days (day 0, 1, 2, 16) in which they visited context-A. Therefore, revisiting context-A at days 2 and 16 may generate a fear extinction effect according to trace dominance theory [43]. It is well-accepted that extinction memory is a new memory that coexists and competes with the original fear memory [44]. In fact, compared with Res-group, we found that female rats in the PBM-group exhibited less freezing response both near the end (4–5 min) of the contextual memory test in context-A (*F*_1, 20_ = 6.087, *P* = 0.023), and throughout the following cued memory test in context-B (*F*_1, 20_ = 10.106, *P* = 0.005) (Fig. 3B). These results suggest that the PBM-treated rats have a greater contextual memory extinction towards context-A. As compared with the rats in Res-group, the much lower freezing baseline (Fig. 3B, 0–1.5 min of cued memory test before tone presentation) suggests that PBM-treated rats can better differentiate the safe context-B during their first visit. It is unlikely that, at the end of the contextual memory test, the decreased freezing behavior in PBM-treated female rats compared to Res-group is a phenomenon of contextual amnesia (or forgetting). This is because during the 3^rd^ minute of the context memory test session, both groups exhibit their highest peak of freezing behavior but lack group differences (*t* (20) = 1.465, *P* = 0.161). In males, both contextual and cued memories were intense (Fig. 3C), and PBM had no treatment effects.

### PTSD-like symptoms and comorbidities in rats are prevented by early PBM interventions

**Fig. 4:**
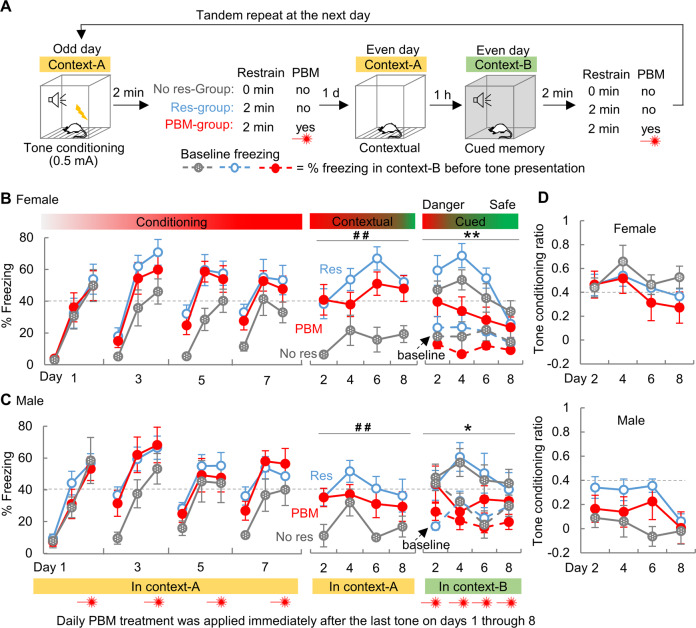
PBM early interventions inhibit trauma-associated fear in a PTSD model of repeated trauma. **A** Rats were conditioned with mild foot shock on odd days (days 1, 3, 5, 7), followed by memory tests on even days (days 2, 4, 6, 8). PBM was applied immediately after the last tone every day. A group of rats without restraint (No res-group, Females, *n* = 7; Males, *n* = 5) was also included. In both females (**B**) and males (**C**), restraint greatly strengthened the contextual, not cued, memory (No res vs. Res). PBM relieved the intense cued memory in both females (Res, *n* = 9; PBM, *n* = 10) and males (Res, *n* = 12; PBM, *n* = 9). **D** The tone conditioning ratio for both sexes.*P < 0.05, **P < 0.01, two way ANOVA, PBM vs. Res; ^##^*P* < 0.01, two way ANOVA, No res vs. Res. Data are mean ± S.E.M.

Since PBM exhibited treatment effects when applied either in the consolidation window or after memory recall, we next investigated whether PTSD**-**like symptoms and comorbidities can be prevented by early PBM intervention. We established a model for complex PTSD, a psychological disorder that develops after an individual experiences prolonged and repeated trauma [45]. Rats were repeatedly conditioned (0.5 mA) on odd days, followed by repeated memory tests (contextual followed by cued 1 h later) on even days as reminders (Fig. 4A, 8 days in total). PBM was applied daily, immediately after every fear conditioning and cued memory test. Consistent with the findings in Fig. 1, restraint applied to the rats in Res-group (Fig. 4) dramatically strengthened contextual memory, as compared with the No res-group. This suggests that immediate restraint boosts hippocampal engagement in contextual encoding. Strikingly, compared with the Res-group, we found that both sexes of the PBM-group exhibited less freezing to cue (Males, *F*_1, 19_ = 4.399, *P* = 0.039; Females, *F*_1, 17_ = 14.139, *P* < 0.001), not context (Males, *F*_1, 19_ = 2.100, *P* = 0.151; Females, *F*_1, 17_ = 2.249, *P* = 0.139) (Fig. 4B, C). To be noted, the cued memory tests performed in context-B on even days, wherein rats repeatedly hear the conditioned tone but never receive foot shocks, could generate a greater extinction effect to context-B, according to trace dominance theory [43]. As such, the abated freezing behavior during the later days of cued memory testing suggests PBM-treated rats can efficiently discriminate “safety” from “danger.” Importantly, after a long interval of three weeks, the PBM-treated rats developed no symptoms or comorbidities of PTSD, while rats in Res-group did (including anxiety, depression, cognition, and social interaction deficits, illustrated in Supplementary Fig. S3). In addition, PBM-treated male rats gained body weight over the days of repeated fear condition training, while treatment control rats (Res-group) lost weight (Supplementary Fig. S4). These results indicate that PBM treatment promotes the correct appraisal of safety during the period of repeated trauma and prevents the development of PTSD-like symptoms and comorbidities in the long run.

### Fear extinction is facilitated by early PBM interventions

**Fig. 5:**
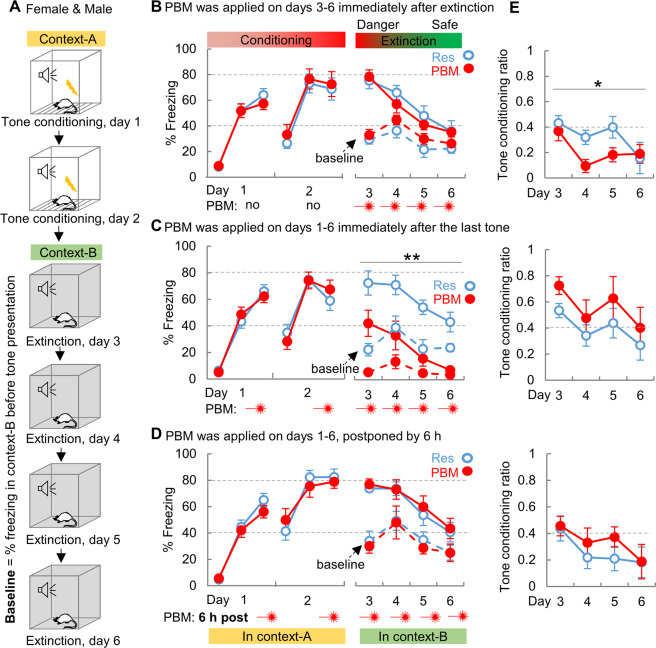
Fear extinction is facilitated by PBM early interventions. **A** Rats were conditioned on days 1–2 in context-A, followed by extinction training on days 3–6 in context-B. **B** PBM exhibited no treatment effect on fear extinction when PBM was applied immediately after extinction training (*n* = 10 each). **C** Extinction was facilitated when PBM was administered immediately after conditioning on days 1 and 2 (Res & PBM, *n* = 9 each). **D** This facilitation effect disappeared when PBM was postponed to 6 h after the last tone (*n* = 10 each). **E** PBM decreased tone conditioning ratio when PBM was applied immediately after extinction training (*F*_1, 18_ = 4.962, *P* = 0.029). **P < 0.01, two way ANOVA, PBM vs. Res. Data are mean ± S.E.M.

To test whether PBM improves fear extinction, rats were conditioned on days 1-2 in context-A, followed by extinction training on days 3–6 in context-B (Fig. 5A, no sex difference was observed; data from females and males were pooled together). Two days of conditioning training was selected to induce an intense cued memory before extinction training. PBM applied immediately after each extinction training (Fig. 5B) did not result in a treatment effect on cued memory (*F*_1, 18_ = 0.572, *P* = 0.459). Interestingly, when PBM was applied immediately after conditioning on day 1–2 (Fig. 5C), a greatly reduced percentage of freezing to tone (*F*_1, 16_ = 27.465, *P* < 0.001) and less intense baseline freezing (*F*_1, 16_ = 52.499, *P* < 0.001) were observed in the PBM-group, compared to Res-group. Notably, even before extinction training on day 3, PBM-treated rats had less baseline freezing (*P* < 0.001, post hoc pairwise comparison) and freezing to tone (*P* = 0.02, post hoc pairwise comparison). This result suggests that PBM-treated rats correctly discriminated the neutral context-B from the “dangerous” context-A during their first visit to context-B on day 3. Moreover, when the PBM application was postponed to 6 h (Fig. 5D) and applied outside of the consolidation window, the treatment effect disappeared (*F*_1, 18_ = 0.250, *P* = 0.623). These findings indicate that PBM is generating a specific effect on the memory consolidation process and that early intervention immediately after foot shock is necessary to maximize the extinction facilitation effect of PBM. It is worth pointing out that the PBM treatments applied immediately after extinction training can also facilitate fear extinction, as PBM-treated rats exhibited a significantly lower tone conditioning ratio on days 4 and 5 (Fig. 5E top panel).

### The hippocampus and amygdala respond to PBM treatment

To explore the cellular and molecular changes triggered by PBM, ERK phosphorylation (pERK) [46] was selected as a marker for monitoring changes in neuronal activity. As shown in Supplementary Fig. S5, PBM increased pERK in both the hippocampus and the amygdala. Immunostaining further confirmed that the increased pERK observed in the hippocampus was co-localized with low-parvalbumin (PV) stained cells. This result suggests that PBM treatment is generating a specific effect on this population of inhibitory neurons in the hippocampus.

## Discussion

Approaches to erase maladaptive fear memory, especially cued hypermnesia, are currently the primary focus in developing treatments for PTSD [47–49]. Although cued hypermnesia is a major symptom of PTSD, another coexisting symptom, contextual amnesia, has been somewhat overlooked [5]. Contextual memory contains spatial and temporal information about a traumatic event and provides reference points to make conscious judgments of whether the current combination of context and cue are dangerous or safe [5, 14]. Here we report that PBM treatment applied after rats experience a traumatic event can boost ATP production in the hippocampus, protect contextual memory from impairment, increase contextual discrimination of a “safe” context, facilitate fear extinction, and, finally, prevent the development of PTSD-like symptoms and comorbidities (outlined in chronological order in Supplementary Fig. S6).

It has been shown that using a tone-shock unpairing protocol (in mice using a 65 dB tone) induced concomitant contextual amnesia with cued hypermnesia [1]. Meanwhile, we found that a tone-shock pairing protocol (in rats with 90 dB tone) can also induce PTSD-like memory impairments, and that the time window for the occurrence of cued hypermnesia lagged behind contextual amnesia. In our experiment setting, contextual amnesia was observed when the contextual memory was tested for the first time (Fig. 1), but cued hypermnesia was observed when the cued memory was tested more than one time (Figs. 3, 4). Throughout this study, the use of the tone-shock pairing procedure implies that the combined presentation of conditioned context and cue during memory testing is the correct predictor for the upcoming foot shock. Therefore, both the memory to context-A and tone could be considered adaptive fear responses. However, when the same tone-shock pairing paradigm was tandemly replicated many times (Fig. 4) or when the tone was repeatedly presented in extinction context (Fig. 5), the tone in context-B predicted the absence of foot shock and indicated a “safe” signal instead. Therefore, a more persistent freezing response to tone specifically on the later rounds of cued memory testing can be considered as cued hypermnesia and a maladaptive fear response. To be noted, this type of cued hypermnesia is associated with a learning deficit of fear extinction to the simple tone. Indeed, as illustrated in Fig. 4, this was validated by the great difference in % freezing between Res- and PBM-group on days 4 and 6 of cued memory testing. Importantly these two groups have divergent phenotypes on whether they develop PTSD-like symptoms and comorbidities (Supplementary Fig. S3). These findings demonstrate that the cued hypermnesia we observed is maladaptive and psychopathological.

Similar to the cue-triggered involuntary memory recall present in PTSD patients, every memory recall event occurs under a specific contextual background. Cued memory testing in animals involves presenting a conditioned tone in a context that is different from the conditioned (or danger-associated) context. When used as a measurement for cued memory, the percentage of freezing to tone has two components, a freezing response to the background context before tone presentation (baseline or pre-tone) and the freezing response to the conditioned tone [38]. In our results, PBM did not exhibit a treatment effect on the tone conditioning ratio (Figs. 1E, 3D, and 4D), with the exception of the fear extinction results (Fig. 5E). The central unifying effect of PBM we observed between experiments is that PBM-treated rats always display a lower fear response to the background context (Figs. 3B, 4B, and 5C), compared with Res-group. The decreased freezing baseline implies a precise contextual memory at the beginning several minutes of each test, which can later facilitate context discrimination and fear extinction.

Numerous evidence has confirmed that physical treatment can generate specific effects on memories if the treatment is applied at special time windows. For example, electroconvulsive shock applied at the consolidation and reconsolidation windows can produce retrograde contextual amnesia [50, 51]; deep-brain stimulation of the entorhinal area during a learning phase can enhance memory [52]; exposing rats to a magnetic field can impair memory acquisition and consolidation for contextual fear memory [53–55]; and transcranial magnetic stimulation, when applied after cue, can produce a reemergence of latent short-term memory in human subjects [56]. Here we report that PBM treatment can prevent contextual amnesia if PBM is applied in the consolidation window after rats experience a high threat traumatic event. It is worth noting that PBM can prevent cued memory from intensifying if PBM is applied after memory recall (Fig. 3). This finding broadly extends the limited treatment window of PBM beyond consolidation. For example, in the clinical setting, PBM could be applied immediately after the PTSD patient has involuntarily retrieved the traumatic memory. As adjuvant therapy during exposure psychotherapy for PTSD, PBM can be applied immediately after the fear memory has been recalled and may help prevent patient dropout because of excessive fear during psychotherapy [57].

Freezing behavior is a natural defensive response in rodents and does not unequivocally prove that a rat is subjectively experiencing a feeling of fear. For example, unlike males, female rats may use “darting” (non-freezing behavior) as an active fear response [58]. In addition, freezing behavior could be affected by factors unrelated to fear, such as changes in sensory input (vision, audition, pain, etc.) or mobility [58, 59]. Therefore, the effect we observed might be non-specific. However, we found that PBM-treated rats from both sexes (data from males may lack the “darting” artifact found in females) displayed a decreased freezing response to the “safe” context and tone (Figs. 3, 4, and 5), but exhibited an increase of freezing behavior to the “danger” context (Fig. 1), compared with treatment control rats. This diverse effect suggests that PBM-treated rats can better differentiate the “safe” context from the “dangerous” one. In addition, we have monitored body weight loss as another parameter for measuring the general pathology from stress. We found that, during the days of repeated fear conditioning testing (Supplementary Fig. S4), males of PBM-treated rats did not lose, but instead gained, body weight at almost the same rate as Naïve-group rats that did not receive any fear conditioning training. Together with the data indicating the absence of other PTSD-like symptoms and comorbidities in PBM-treated rats (Supplementary Fig. S3), these findings support that freezing behavior does reflect a fear state in rats and that PBM-treated rats have less psychopathological fear but a more precise contextual fear memory, which indicates improved context discriminability.

It is possible that PBM may have no effect on the memory of the fear conditioning event and instead may only generate beneficial effects relating to the stress from restraint. Since rats were restrained during PBM treatment and both restraint and fear conditioning training generate stress, it is difficult to dissect whether PBM has an effect specifically on the stress from restraint, fear conditioning training, or the contextual memory itself. For example, although we demonstrated that the beneficial effects of PBM were compromised when PBM was applied 6 h after conditioning (Supplementary Fig. S2), and this finding suggests that PBM may target memory consolidation, there is an important limitation on the interpretation of this finding. Because PBM must be applied during a restraint stress episode, we cannot rule out whether the missing beneficial effect is due to the 6 h shift of PBM per se or to the shift of restraint stress. Nevertheless, we found that PBM-treated male rats displayed a significantly attenuated freezing response during cued memory tests compared with No res-group (*F*_1, 13_ = 4.454, *P* = 0.038, Fig. 4B). This result suggests that PBM generates beneficial effects beyond the stress from restraint. To be noted, (I) our results and others [1, 18] demonstrate that restraint stress applied immediately after fear conditioning is necessary for inducing a PTSD-like phenotype in rodents, (II) the same set of stress hormones, norepinephrine, and glucocorticoids, are released regardless of the source of stressful events, (III) these stress hormones influence the memory consolidation process for the stressful event [4].

We observed a significant sexual dimorphism during the contextual memory test. Under high-stress conditions with a strong foot shock, male rats exhibited a much higher freezing response to the context compared with females (Figs. 1 and 3). In contrast, such sexual dimorphism on contextual memory was not observed when the stress intensity was low. These findings imply that males and females have different memory processing patterns for contextual information for high threat events. Together with the higher basal level of corticosterone in females’ hippocampus and plasma (Fig. 2), these findings may explain why females are more sensitive to stress and have a higher risk of developing PTSD [36]. While we did observe gender differences in PBM-treated animals in this study, we believe that they are associated with PTSD psychopathology rather than the treatment itself. PTSD is well known to affect women differently, both in rates and symptoms, than men. Meanwhile, we cannot completely rule out sex differences in PBM response, because our previous studies did not reveal any gender differences in PBM treatment of uninjured rats or in multiple models of brain injury.

The molecular mechanism of contextual amnesia is an interesting research area that, unfortunately, is still underdeveloped. Early work has shown that the combination of a relatively high stressful situation and corticosterone injection is associated with hypofunction of the hippocampus in context coding and appears as the primary cause of contextual amnesia [1, 38]. This concept is further supported by a recent study that reports optogenetic activation of the dorsal hippocampus prevents the occurrence of contextual amnesia [38]. To explore whether the hippocampus responds to PBM treatment, we compared ERK phosphorylation levels between Res- and PBM-group (Supplementary Fig. S5) without the interference of fear conditioning training [60]. We found that PBM treatment can induce transient activation of ERK phosphorylation in the hippocampus for at least 30 min after PBM treatment. Although PBM does not affect corticosterone release in our results, PBM can increase ATP production nearly twofold 20 min after fear conditioning (Fig. 2). Experiments using different tissues show that PBM’s ATP boosting effect can last up to 3 h [28, 29], which covers the most critical part of the memory consolidation phase. As a direct energy supplier, ATP can induce long-term potentiation and facilitate synaptic transmission [61, 62]. This may explain the consolidation protection effect from PBM. To be noted, these results did not provide causal relationships between PBM and prevention of contextual amnesia at the molecular level; nevertheless, these findings give a hint on how a therapeutic approach may reverse contextual amnesia.

In this preclinical study, we demonstrate an early intervention treatment can prevent the development of PTSD-like symptoms and comorbidities in rats. Because PBM is applied to the skull and delivers light to the whole brain, which brain regions benefit from PBM is worthy of further investigation. Since human skulls are much thicker than rats, the limited penetration depth of PBM in the human brain could be a barrier for translational application. Future work should focus on optimizing the treatment parameters for human application to assist those coping with catastrophe and reduce their persistent psychopathological fear.

## Supplementary information

Supplementary Information
