# Supplementary material for: Photobiomodulation prevents PTSD-like memory impairments in rats
Source: Mol Psychiatry. 2021 Apr 15;26(11):6666–79. doi: 10.1038/s41380-021-01088-z (PMC8760076; doi:10.1038/s41380-021-01088-z)
Supplement: Supplementary file 1 — Supplementary Information [file 41380_2021_1088_MOESM1_ESM.pdf]

Supplementary Information

Supplementary Materials and Methods

6 Supplementary Figures

1 Supplementary Table

Supplementary Materials and Methods

Behavior tests for PTSD symptoms and comorbidities. The following behavior tests were performed three weeks after the repeated fear conditioning test. According to the different stress levels caused by different behavior tests, the behavioral tests were performed in a sequence order from low stress to high as indicated below (one test/day except novel object test).

Open field arena. Exploratory motor activity was tested in an open field arena as a beginning control for the following behavior tests for PTSD symptoms and comorbidities. The open field arena (50 × 50 × 50 cm) was made of black laminated particle boards. Rats were placed in the open field arena and allowed to explore for 5 min under 100 lux illuminations. The total travel distance in 5 min was plotted and analyzed by "ANY-Maze" software.

Novel object test. Long-term memory was tested in the novel object test. Rats were exposed to two identical objects for 10 min (exposure session) in the open field arena described above. After a 24 h interval, rats were exposed to the familiar object and a novel object (test session) for 10 min. Two golf balls were glued together and was used as a novel object (diameter 4 cm, height 7.5 cm); a Lego brick cuboid (3.2 × 3.2 × 7.5 cm) was used as a familiar object. The preference (≥ 50 % as memory) for the novel object was calculated by percent of exploration time spent investigating the novel object (golf) divided by total exploration time (golf + lego). The exploration time was defined by rats approaching its nose within 2 cm of the object tracing by "ANY-Maze" software.

Sociability and social novelty test. Three chambers, each sized 33 × 45 × 45 cm, were used. Rats were first placed in the middle chamber for 5 min for habituation, then the door was opened to allow the rat to

explore the other two chambers on opposing sides (one was empty and the other contained Rat 1) for 8 min. Ratio of time spent investigating the chamber with Rat 1/ (Rat 1 + empty) was plotted as an indicator of sociability, the preference for the chamber with rat vs. empty. At 4 h later, a new rat (Rat 2) was introduced into the empty chamber, and the rat being tested was placed in the middle chamber and tested for 8min. Ratio of time spent investigating Rat 2/ (Rat 1 + Rat 2) was plotted as an indicator of social novelty, the preference of the chamber with new rat vs. familiar rat.

Elevated plus maze. Anxiety levels of rats were tested in an elevated plus maze. The "+" shape maze was a made of black laminated particle boards. The width of the track was 10 cm. The length of both closed and open arms were 110 cm with an intersection in the middle. The closed arms were surrounded by 45 cm high walls along the edge of the track. The maze was elevated 50 cm above the floor. A rat was allowed to explore the maze for 5 min. The time of the rat visiting the open arms was counted for evaluating anxiety. Open arm entries were defined as all four paws entering the open arms for at least one second.

Freezing to unexpected noise. The rat was placed in the arena (open field test chamber) for 90 s and then presented with three 30 s white noise (70 dB) every 2 min (the same protocol as cued memory test but with a different sound). Baseline (% freezing before white noise) and the average of % freezing to the 30 s white noise was plotted.

Re-experiencing. Four weeks after the repeated fear conditioning test, the rat was placed in the conditioning chamber (context-A) for 6 min following the same protocol as the conditioning session with tone but not foot shock. Percent freezing time in each 2 min session was plotted.

Forced swimming test. Depression levels of rats were tested in a tank (sized 30 × 37 × 56 cm) filled with water to 36 cm height. Rats were allowed to swim for 5 min. The immobility time was measured for the level of depression. Immobility behavior (no noticeable movement for ≥ 1 s) was analyzed by "ANY-Maze" software. Immobility was defined as a rat not moving its four paws for at least one second.

Body weight. One day before fear conditioning training, the body weight of each rat was measured and normalized as 100 %. Body weight was measured at day 4, 7 and 10 for monitoring the total loss of body weight during the repeat fear conditioning test and the extinction test.

Western blot. Naïve rats were treated with either PBM or restraint for 2 min. At 0.5 and 2 h later, rats were anaesthetized by isoflurane and the brain was immediately harvested and kept in ice cold ACSF. A 500 µm thickness of coronal brain slices were made on a vibrating slicer (Leica VT1200S) in the solution of ice cold ACSF. The prefrontal cortex (Bregma 2.7 mm to 2.2 mm), dorsal hippocampus, and amygdala (Bregma -2.3 mm to -2.8 mm) were isolated and put into an ice-cold RIPA buffer (Thermo Scientific), which was supplemented with a protease inhibitor cocktail (Sigma-Aldrich) and a phosphatase inhibitor cocktail (Thermo Scientific) as previously described. After tissue homogenization and sonication, protein lysates were isolated by centrifugation. The supernatants were collected and incubated with the Pierce Lane Marker Reducing Sample Buffer (Thermo Scientific) for 3 min at 95 °C. The protein samples (20 µg total proteins per well) were run on 4-20 % SDS polyacrylamide gel and then transferred onto PVDF membrane. After washing with PBST, the membranes were blocked for 1 h at 23 °C in a PBST blocking solution (PBST + 2 % bovine serum albumin), and then incubated with antibodies against pERK and panERK (#4370 and #4695, Cell Signaling technology, Topsfield, MA, USA) overnight at 4 °C. The

membranes were washed with PBST and incubated with HRP-conjugated anti-IgG (1:10,000; Bio-Rad, Portland, ME, USA) for 1 h at 23 °C. Bands were visualized with a chemiluminescence detection solution (Thermo Scientific).

Immunostaining of pERK. Naïve rats were treated with PBM or restraint for 2 min. At 30 min and 2 h later, rats were anaesthetized by isoflurane and fixed by cardiac perfusion with 4 % neutral buffered formalin. The harvested brains were stored in 4 % neutral buffered formalin for 1 day at 23 °C. Coronal brain slices of the dorsal hippocampus were made with a 50 µm thickness on a vibrating slicer (Leica VT1200S, Topsfield, MA, USA). Brain slices (Bregma -2.3 mm to -2.8 mm) were washed with PBS containing 0.1 % Triton X-100 (referred to as PBST) twice for 5 min each, and then blocked by 4 % normal goat serum in PBST for 1 h at 23 °C. The primary antibodies against pERK) and parvalbumin (PV) (#PV235, Swant Inc, Marly, Switzerland) were diluted at 1:300 in PBST containing 4 % normal goat serum. After incubation with the primary antibodies overnight at 4 °C, the slices were washed with PBST and incubated with secondary antibodies conjugated with Alexa 488 and Alexa 568 (Thermo Scientific) (1:300 in PBST) for 2 h at 23 °C. After being washed with PBST, the slices were mounted with ProLong Diamond Antifade Mountant with DAPI (Life Technologies, Guilford, CT, USA). The images were scanned by confocal microscope (Zeiss LSM 700, Peabody, MA, USA). The percentage of pERK positive cells were calculated by the numbers of pERK positive cells divided by low-PV staining cells per image.

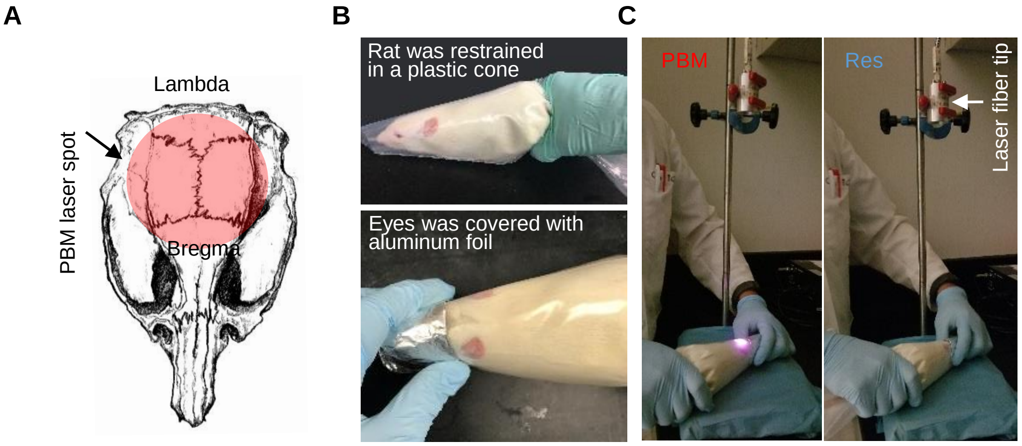

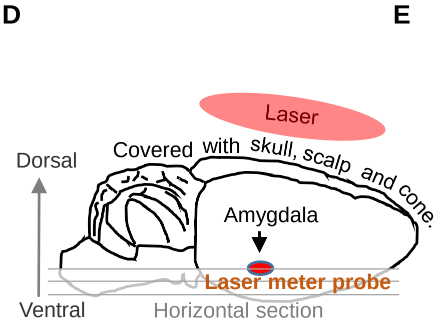

E

Wavelength	808 nm
Power	350 mW/cm², scalp;<br>25 mW/cm², cortex;<br>12 mW/cm², dorsal hippocampus;<br>3 mW/cm², amygdala.
Irradiation time	2 min
Beam area	1.5 cm²
Pulse parameters	continuous wave
Anatomical location	on skull
Number of treatments	one or multiple
Treatment interval	after each memory recall

Figure S1. The detailed procedure for applying PBM and restraint. a Red circle shadow denotes the area of PBM application on the rat skull. **b** Rat under restraint with eyes covered with aluminum foil. **c** Rat receiving PBM treatment (left panel) and control treatment (right panel). **d** The setup for measuring beam intensity penetrated to amygdala. **e** Laser beam parameters.

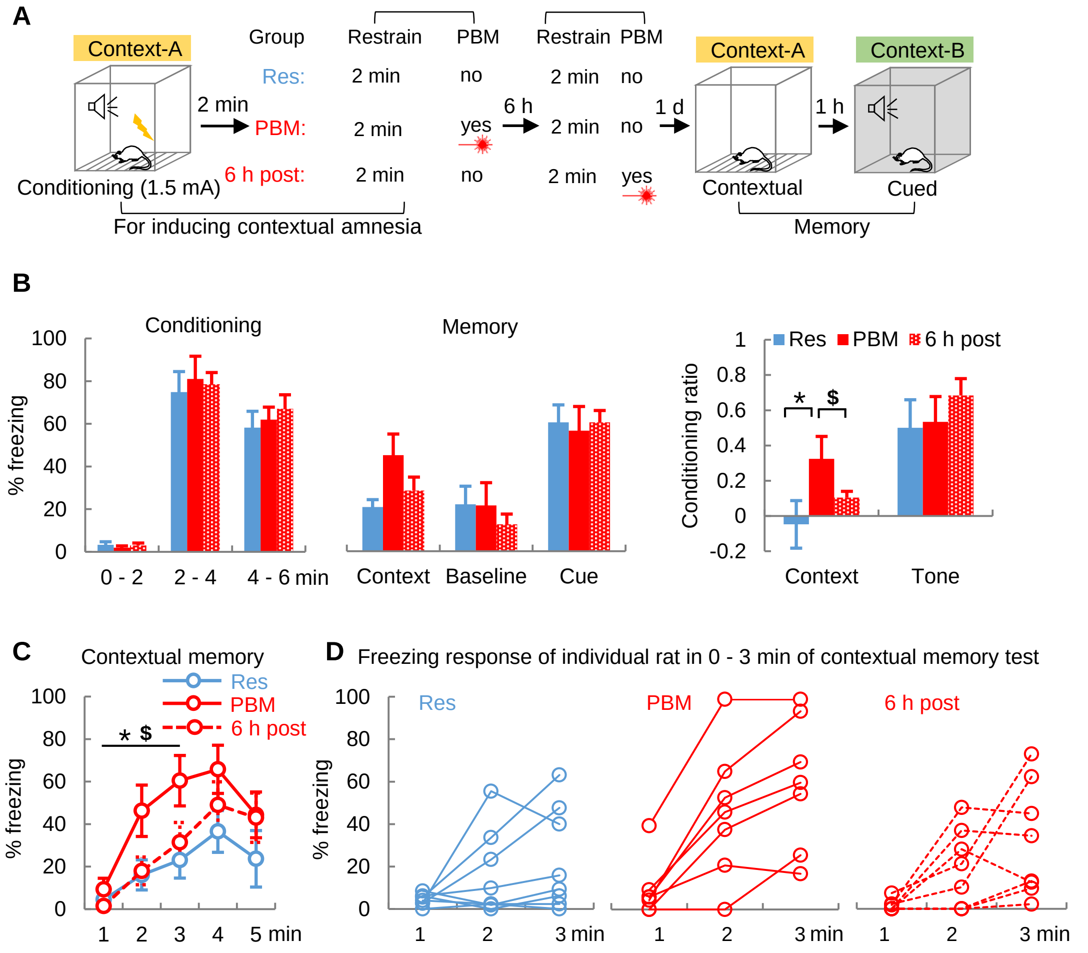

Figure S2. The protection effect of PBM is compromised when PBM is postponed to 6 h outside of consolidation window. a The detailed procedure for inducing contextual amnesia and applying PBM. Rats (females) were subjected to the same stress conditions that can induce contextual amnesia as in Fig. 1. At 6 h after the first round of restraint, rats received another round of restraint. Rats who received PBM during the first and second round of restraint were named the PBM-group (*n* = 7) and 6 h post-group (*n* = 8), respectively. Res-group (*n* = 8) were restrained after conditioning but did not receive PBM treatment. **b** The freezing response during conditioning and memory tests. **c** The minute to minute freezing response during the contextual memory test. Note, we used two-way ANOVA with repeat measures and analyzed the first three minutes of freezing response when the rat started to recognize the conditioning chamber and begin freezing. We found that PBM treatment applied immediately after conditioning improved contextual memory (* PBM vs. Res, $F_{(1, 13)}$ = 5.832, P = 0.031), but the protection effect was compromised when PBM was postponed to 6 h ($ PBM vs. 6 h post, $F_{(1, 13)}$ = 4.827, P = 0.047). **d** The freezing response of an individual rat during the first three minutes of the contextual memory test. Compared with Res- and 6 h post-group, rats in PBM-group showed a higher freezing response to the conditioning context. Data with error bars are mean ±S.E.M.

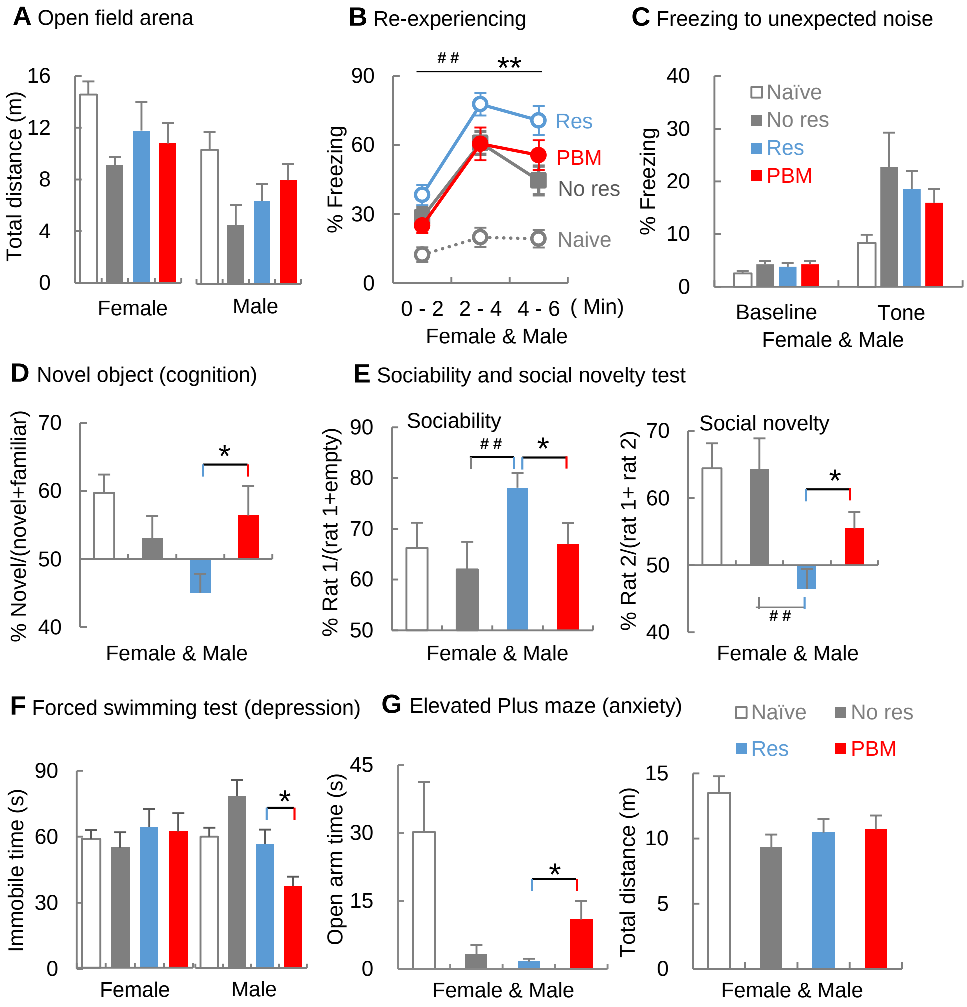

Figure S3. PTSD-like symptoms and comorbidities in rats were prevented by PBM early interventions. a As a beginning control for the following behavior tests, the open field test found no difference in mobility between PBM-group (red column, *n* = 10 & 9 for females and males, respectively) and Res-group (blue column, *n* = 9 & 9). Naïve-group (white) rats never experienced fear conditioning and restraint. No res-group (grey) rats were not restrained after fear conditioning and memory test. **b** Re-experiencing context-A with tone again, rats in PBM-group (*n* = 19 total for both sexes) exhibited less freezing than Res-group (*n* = 15); $F_{(1, 32)} = 5.234$, $P = 0.029$, two-way ANOVA with repeat measures. **c** PBM had no effects on the freezing response to an unexpected white noise. **d** PBM recovered memory deficits in the novel object test (t (27) = 2.162, $P = 0.040$; PBM, *n* = 15 vs. Res, *n* = 14). **e** PBM prevented the abnormal sociability pattern (left panel, t (30) = 2.129, $P = 0.042$, PBM, *n* = 15 vs. Res, *n* = 17) and social novelty deficits (right panel, t (30) = 2.355, $P = 0.025$, PBM vs. Res) which were observed in Res-group. **f** Less immobile time in the forced swimming test indicates that rats (males only) in PBM-group (*n* = 9) exhibited less depression-like behaviors than Res-group (*n* = 12) (t (19) = 2.287, $P = 0.034$). **g** More time traveled in open arms of elevated plus maze suggests that PBM-treated rats (*n* = 25) had less anxiety than Res-group (*n* = 23) (left panel, t (46) = 2.090, $P = 0.042$). As a control, the two groups had similar total travel distances (right panel). * $P < 0.05$, ** $P < 0.01$, PBM vs. Res; $^{##}$ $P < 0.01$, No res vs. Res. Data are mean $\pm$S.E.M.

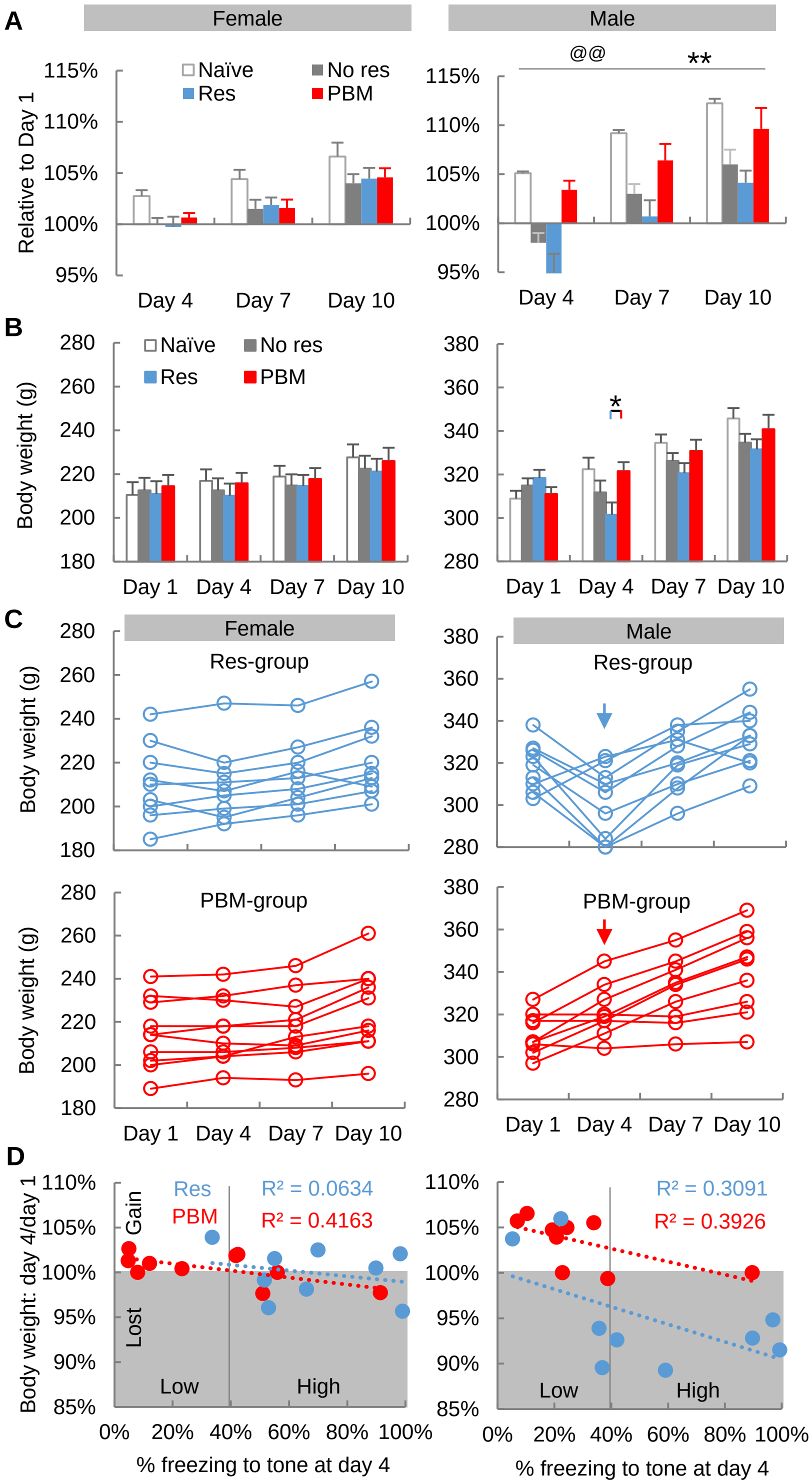

Figure S4. Body weight loss during the days of the repeated trauma was prevented by PBM. a Unlike females, male rats in Res-group lost body weight during the eight days fear conditioning test. PBM prevented the body weight loss in males ($F_{(1, 16)} = 23.68$, $P < 0.001$, two-way ANOVA), but has no effect on body weight in females ($F_{(1, 17)} = 0.139$, $P = 0.711$). Naïve, $n = 6$ & 12; No res = 7 & 6; Res, $n = 9$ & 9; PBM, $n = 10$ & 9 for females & males, respectively. @@ $p < 0.01$, No res vs. Naïve; ** $p < 0.01$, two way ANOVA, PBM vs. Res. **b** The absolute body weight for each group. On day 4, male rats in PBM-group had a higher body weight than Res-group (t (16) = 2.870, $P = 0.011$) . **c** Each line represents the absolute body weight of an individual rat spanning the eight days of repeated fear conditioning training. After two rounds of fear conditioning training at day 4, seven out of nine male rats in Res-group lost body weight (blue arrow). Intriguingly, six out of nine male rats in PBM-group gained body weight (red arrow). Unlike males, females rats in both groups exhibited less fluctuation in body weight changes. **d** The correlation analysis for the body weight status and the corresponding fear response during cued memory test at day 4. Male rats in PBM-group (red dots) had a dramatically lower freezing response to the tone in a “safe” context and increased rate of body weight gain compared with rats in Res-group (blue dots). Dashed line represents the trend line from linear analysis.

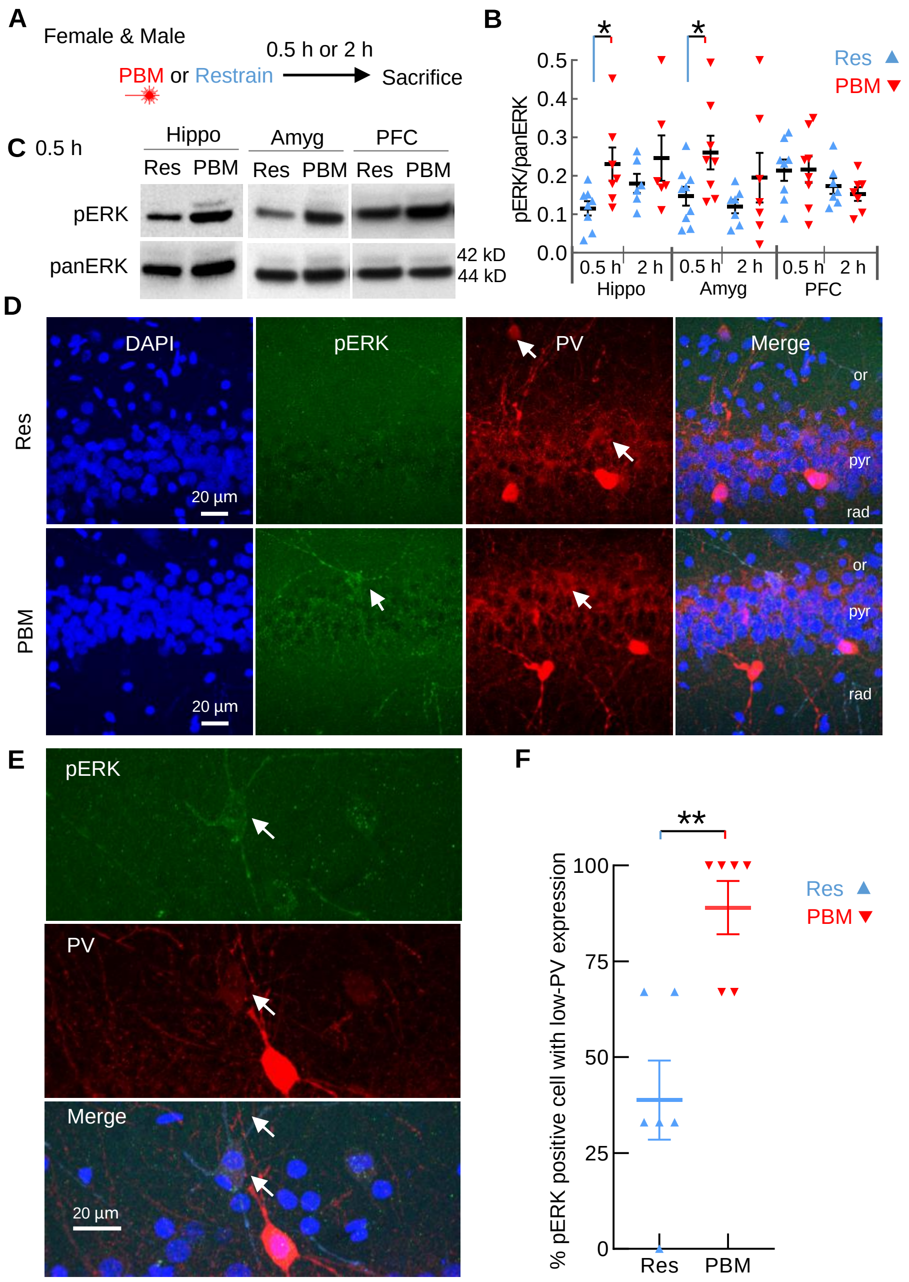

Figure S5. PBM triggers ERK phosphorylation in the hippocampus and amygdala. a Naïve rats were restrained along with or without PBM, and then were sacrificed at 0.5 h and 2 h post PBM. **b** PBM increased ERK phosphorylation in the hippocampus (Hippo, t (13) = 2.550, P = 0.024; Res, n = 8 & PBM, n = 7) and amygdala (Amyg, t (15) = 2.326, P = 0.034; Res, n = 9 & PBM, n = 8) at 0.5 h, not 2 h post treatment (Res, n = 6 for Hippo, n = 7 for Amyg; PBM, n = 6 for both Hippo and Amyg). PBM had no effects on pERK in the prefrontal cortex (PFC, n = 7 each). **c** Representative Western blot images from 0.5 h post PBM in hippocampus CA1 area. **d** Increased pERK originated from low-parvalbumin (PV) staining cells (arrow). Images were taken from CA1. n = 6 slices from 6 rats for each group. **e** Another magnified interneuron (arrow) from PBM group with high pERK and low-PV staining. **f** Cell counting result confirmed that PBM increase the percentage of pERK positive cell with low-PV expression. t (10) = 4.012 , P = 0.002, Res vs. PBM). * P < 0.05, ** P < 0.01, PBM vs. Res, student's t-test. Data are mean ±S.E.M.

PBM early intervention in consolidation window

↓

Increases ATP in the hippocampus following stress (Fig. 2)

↓

Prevents contextual fear memory from impairment (Fig. 1)

↓

Increases the context discrimination of “safe” from “danger” (Fig. 3)

↓

Reduces contextual fear following memory recall (Fig. 4)

↓

Facilitates fear extinction in a “safe” context (Fig. 5)

↓

Prevents the development of PTSD-like symptoms and comorbidities in rats (Fig. S3 and S4)

Figure S6. The working flow of the benefiting effect from PBM early intervention.

Supplementary Table 1

	Gender	Stress mA+min	PBM	Group name	*n* =	Statistical methods used for the comparison between two groups: two way ANOVA and *t*- test.							
Fig. 1 contextual amnesia.	Female	0.5+0	No	No res	7		No res	#					
		0.5+2	No	Res	9		*t* -test	Res		$			
		0.5+2	Yes	PBM	10			*t* -test	PBM				
		1.5+2	No	Res	11			*t* -test		Res	*		
		1.5+2	Yes	PBM	11					*t* -test	PBM		
	Male	0.5+0	No	No res	5		No res	#					
		0.5+2	No	Res	14		*t* -test	Res					
		0.5+2	Yes	PBM	15			*t* -test	PBM				
		1.5+2	No	Res	9			*t* -test		Res		$	
		1.5+2	Yes	PBM	9					*t* -test	PBM		
		1.5+10	No	Res	8					*t* -test		Res	*
		1.5+10	Yes	PBM	8							*t* -test	PBM
Fig. 2 stress hormones and ATP	Female	0.0+0	No	Naïve	4	Naïve	@						
		1.5+0	No	No res	4	*t* -test	No res	#					
		1.5+2	No	Res	9		*t* -test	Res	*				
		1.5+2	Yes	PBM	9			*t* -test	PBM				
	Male	0.0+0	No	Naïve	4	Naïve	@						
		1.5+0	No	No res	4	*t* -test	No res	#					
		1.5+10	No	Res	10		*t* -test	Res	*				
		1.5+10	Yes	PBM	10			*t* -test	PBM				
Fig. 3 memory recall	Female	1.5+0	No	No res	8		No res	*t* -test					
		1.5+2	No	Res	10		ANOVA	Res	*t* -test				
		1.5+2	Yes	PBM	12			ANOVA	PBM				
	Male	1.5+0	No	No res	8		No res	*t* -test					
		1.5+2	No	Res	8		ANOVA	Res	*t* -test				
		1.5+2	Yes	PBM	8			ANOVA	PBM				
Fig. 4, S3 and S4 repeated trauma	Female	(0.0+0)×4day	No	Naïve	6	Naïve	*t* -test						
		(0.5+0)×4day	No	No res	7	ANOVA	No res	*t* -test					
		(0.5+2)×4day	No	Res	9		ANOVA	Res	*t* -test				
		(0.5+2)×4day	Yes	PBM	10		ANOVA	ANOVA	PBM				
	Male	(0.0+0)×4day	No	Naïve	12	Naïve	*t* -test						
		(0.5+0)×4day	No	No res	5	ANOVA	No res	*t* -test					
		(0.5+2)×4day	No	Res	12		ANOVA	Res	*t* -test				
		(0.5+2)×4day	Yes	PBM	9		ANOVA	ANOVA	PBM				
Fig. 5 extinction	Female & male	(0.5+0)×2day	No	Res	10			Res	*				
		(0.5+0)×2day	Yes	PBM d3-d6	10			ANOVA	PBM				
	Female & male	(0.5+2)×2day	No	Res	9			Res	*				
		(0.5+2)×2day	Yes	PBM d1-d6	9			ANOVA	PBM				
	Female & male	(0.5+2)×2day	No	Res 6h post	10			Res					
		(0.5+2)×2day	Yes	PBM 6h post	10			ANOVA	PBM				
Fig. S2 PBM 6h postphone	Female	1.5+2+2	No	Res	8			Res	*				
		1.5+2+2	Yes	PBM	8			*t* -test	PBM	$			
		1.5+2+2	Yes	6 h post	8				*t* -test	6 h			
Fig. S5 pERK	Female & male	0.0+2	No	Res 0.5h	9			Res	*				
		0.0+2	Yes	PBM 0.5h	8			*t* -test	PBM				
	Female & male	0.0+2	No	Res 2h	7			Res					
		0.0+2	Yes	PBM 2h	6			*t* -test	PBM				
**Total**				**47 groups**	**406**	@ # * $ denotes significant difference, $P < 0.05$							
